# Agreement of pCO_2_ in venous to arterial blood gas conversion models in undifferentiated emergency patients

**DOI:** 10.1186/s40635-023-00564-w

**Published:** 2023-11-21

**Authors:** Matthias Jörg, Malin Öster, Jens Wretborn, Daniel B. Wilhelms

**Affiliations:** 1 Department of Emergency Medicine, Sundsvall Regional Hospital, Region Västernorrland, Sundsvall, Sweden; 2 Department of Biomedical and Clinical Sciences, Linköping University, 58185 Linköping, Sweden; 3 Department of Emergency Medicine, Local Health Care Services in Central Östergötland, Region Östergötland, Linköping, Sweden

**Keywords:** Blood gas, Conversion, pCO_2_, Arterial, Venous, Emergency department

## Abstract

**Background:**

Venous blood gas sampling has replaced arterial sampling in many critically ill patients, though interpretation of venous pCO_2_ still remains a challenge. Lemoël et al., Farkas and Zeserson et al. have proposed models to estimate arterial pCO_2_ based on venous pCO_2_. Our objective was to externally validate these models with a new dataset. This was a prospective cross-sectional study of consecutive adult patients with a clinical indication for blood gas analysis in an academic emergency department in Sweden. Agreement of pairs was reported as mean difference with limits of agreement (LoA). Vital signs and lead times were recorded.

**Results:**

Two hundred and fifty blood gas pairs were collected consecutively between October 2021 and April 2022, 243 valid pairs were used in the final analysis [mean age 72.8 years (SD 17.8), 47% females]. Respiratory distress was the most common clinical indication (84% of all cases). The model of Farkas showed the best metrics with a mean difference between estimated and arterial pCO_2_ of − 0.11 mmHg (95% LoA − 6.86, + 6.63). For Lemoël the difference was 2.57 mmHg (95% LoA − 5.65, + 10.8), Zeserson 2.55 mmHg (95% LoA − 7.43, + 12.53). All three models showed a decrease in precision in patients with ongoing supplemental oxygen therapy.

**Conclusion:**

Arterial pCO_2_ may be accurately estimated in most patients based on venous blood gas samples. Additional consideration is required in patients with hypo- or hypercapnia or oxygen therapy. Thus, conversion of venous pCO_2_ may be considered as an alternative to arterial blood gas sampling with the model of Farkas being the most accurate.

**Supplementary Information:**

The online version contains supplementary material available at 10.1186/s40635-023-00564-w.

## Take home message

Arterial pCO_2_ may be accurately estimated in most ED patients based on venous blood gas samples using a mathematical model. Conversion of venous pCO_2_ may be considered a viable alternative to measuring pCO_2_ by arterial blood gas sampling in most situations in the ED. Additional consideration is required in patients with hypo- or hypercapnia or oxygen therapy.

## Background

Blood gas analysis is a crucial tool in the assessment of critically ill patients in the emergency department (ED) [1].

The reference standard for blood gas analysis has traditionally been arterial blood gas (ABG) sampling. However, ABG sampling is associated with considerable pain for the patient, carries a small risk of vascular complications and chronic neurologic injury related to the arterial puncture [2, 3]. Furthermore, ABG sampling requires skilled staff which may be limited in certain situations or regions of the world, e.g., middle and low-income countries [4].

Consequently, venous blood gas (VBG) sampling has replaced arterial sampling in many situations over the last decade. It has been shown that the venoarterial differences in pH, HCO_3_ and base excess lack clinical significance [1, 5].

However, the reliability of variables defining the respiratory components of a venous blood gas is still debated [6, 7]. Venous partial pressure of oxygen (pvO_2_) and oxygen saturation in venous blood (SvO_2_) are strongly dependent on oxygen uptake in the periphery and by that unreliable as a marker for pulmonary oxygenation [8].

The partial pressure of carbon dioxide (pCO_2_) represents one of the most important clinical variables in respiratory evaluation of critically ill patients in the ED [9]. It has been shown to be unreliable in venous blood samples, however, several different approaches for conversion between venous and arterial values to mitigate this have been suggested [5, 10]. Based on 129 ED and intensive care patients, Zeserson et al. proposed a linear correlation model for conversion between venous and arterial pCO_2_ using simple subtraction of a static value [11]. This linear conversion, which is commonly used in practice, has shown considerable variation in different patient populations which questions its reliability [12–15].

Another approach is adaptive conversion that factors in SvO_2_ as an indirect marker of oxygen uptake and strain in peripheral tissue. Two similar models using this method have been derived by Lemoël et al. and Farkas, respectively [16, 17].

The article describing Farkas’ model has never been published in a peer-reviewed journal, but the manuscript was made freely available online [17]. His proposed model was developed by using four different datasets from at least three different countries and Farkas himself was not involved in the data collection [17]. Lemoël’s model, in contrast, builds on only one dataset acquired by the same group with the purpose to develop a conversion model [16].

Although the results of all three models were promising in the derivation datasets, none of them has been validated externally, nor have they ever been systematically evaluated in everyday clinical practice in an ED. Furthermore it remains unclear how confounding factors relevant for conversion reliability, e.g., difficult blood gas sampling resulting in time delay and ingression of room air or rapidly changing physiological states due to resuscitation efforts, affect the suggested models [18, 19]. The extrapolation of “true” arterial values by conversion models require both valid and reliable results. In the vast majority of clinical situations, however, an approximate value may be sufficient, as long as the margin of error is narrow and that limitation is known to the clinician.

Thus, external validation of the proposed conversion models for venous to arterial pCO_2_ can further strengthen the role of VBG as an informative alternative to ABG for pCO_2_ evaluation in ED patients, e.g., as a quick estimate outside the ICU guiding emergency physicians and intensivists in the decision on level of care prior to admission or the decision of no escalation of therapy.

### Goals of this investigation

To evaluate existing venous to arterial blood gas conversion models by Lemoël et al., Farkas and Zeserson et al. for the agreement of pCO_2_ in undifferentiated ED patients.

## Methods

### Study design and setting

This was a prospective cross-sectional study of consecutive patients aged 18 years or older. Data collection was done during an 8-week period divided into three blocks between October 2021 and April 2022 in the ED of Linköping University Hospital in Sweden. During each data collection block, an on-site enrollment coordinator facilitated consecutive inclusion between 8 and 2 am daily irrespective of mode of arrival. Outside these hours, enrollment was delegated to the resuscitation team on duty. The coordinator team consisted of eight senior emergency physicians and nurses assigned for screening and enrollment tasks only.

### Selection of participants

We included adult patients (≥ 18 years of age) with an indication for arterial blood gas sampling, specifically evaluation of paO_2_ or pCO_2_, according to triage priority and standard operating procedure (SOP) as assessed by the treating physician.

We excluded children, pregnant women and patients unwilling to participate or unable to understand given oral and written information in Swedish with one exception; patients with the inability to understand the given oral and written information due to an acute critical condition were allowed to be included through deferred consent (< 12 h) or through a physically present relative who did not oppose on research.

### Study protocol and data collection

Informed consent (if possible) was obtained during the initial assessment. Following routine clinical practice, sampling of an arterial blood gas and a venous blood gas was performed simultaneously or as close in time as possible. Sampling sites were the radial artery and peripheral veins on the upper extremity with no demand for ipsilateral sampling given the clinical circumstances. For venous sampling a tourniquet was used. For both venous and arterial sampling, a standard 1 ml pre-heparinized blood gas syringe was used.

Sampling was carried out by physicians or nurses in the treating team. Since delay time from sampling to analysis is a known confounder of pCO_2_, the enrollment coordinator supported the treating teams with sampling and analysis when possible.

The following variables were recorded on the patient worksheet by the enrollment coordinator prior to sampling: chief complaint according to Rapid Emergency Triage and Treatment System (RETTS) [20], the patient's current vital signs (heart rate in bpm, systolic and diastolic blood pressure in mmHg, respiratory rate in bpm, oxygen saturation in %, body temperature in °C), ongoing oxygen treatment (L/min). Immediately after sampling, puncture location, number of attempts and lead times (in minutes) were recorded.

Blood gas analysis was performed using one of three similar blood gas analyzers located in the study ED (all ABL90 Series, Radiometer Medical ApS, Bronshoj, Denmark). The analyzers were calibrated according to the manufacturer’s recommendations and no deviations or technical errors occurred during the study period.

The results of blood gas analysis were extracted from the internal memory of the analyzers.

### Outcome measures

The primary outcome was the agreement of estimated arterial pCO_2_ based on venous blood sampling for the conversion models previously suggested by Farkas, Lemoël et al. and Zeserson et al. [11, 16, 17].

The investigated conversion model formulas below are presented for both mmHg and kPa use. All results related to pCO_2_ are primarily presented in mmHg, for corresponding values in kPa (conversion modifier 0.133) please refer to the supplementary material.

We calculated the estimated carbon dioxide partial pressure (peCO_2_) value from the venous sample using venous carbon dioxide partial pressure (pvCO_2_). For the models of Farkas and Lemoël, we also used venous saturation (SvO_2_). The following formulas were used:

**Table:** 

Farkas model in mmHg peCO_2_ = pvCO_2_ – (0.22 * (93 – SvO_2_))	Farkas model in kPa peCO_2_ = pvCO_2_ – (0.03 * (93 – SvO_2_))
Lemoël model in mmHg peCO_2_ = pvCO_2_ – (0.3 * (75 – SvO_2_))	Lemoël model in kPa peCO_2_ = pvCO_2_ – (0.04 * (75 – SvO_2_))
Zeserson model in mmHg peCO_2_ = pvCO_2_ – 4.8	Zeserson model in kPa peCO_2_ = pvCO_2_ – 0.64

We then calculated the difference between the estimated peCO_2_ and the actual arterial carbon dioxide partial pressure (paCO_2_) of the corresponding arterial sample:

$$\Delta \, = {\text{ peCO}}_{{2}} {-}{\text{ paCO}}_{{2}} .$$

### Study size

A sample size calculation was not done a priori and instead a convenience sample of 250 paired samples was considered reasonable and in line with similar published work [16, 21, 22].

### Analysis

Descriptive data were reported as percentage, mean with standard deviation (SD) or median with interquartile ranges (IQR). To estimate the interval of the differences between measurements, data are shown in the Bland–Altman plot with limits of agreement (LoA) and percentage error (PE) [23]. Logistic regression analysis was performed to assess for confounding factors using age (continuous), supplemental oxygen (yes/no), sex (male/female), systolic blood pressure (continuous), respiratory rate (continuous) and body temperature (continuous) and reported as odds ratios (ORs). Scatter plots with Pearson's correlation coefficient (*r*) were used to depict differences in the models related to time delay between sample occasions. Statistical analysis was performed with R (version 4.1.3) and graphs were rendered with the ggplot2 package (version 3.4.1).

## Results

### Enrollment and study population characteristics

In total, 250 blood gas pairs were collected during three blocks between October 2021 and April 2022. After exclusion of seven pairs due to missing data or consent not provided within 12 h, 243 pairs were used in the final analysis. Mean age was 72.8 years (SD 17.8) and 47% (113/243) were female.

The indications for blood gas sampling were respiratory failure in 204 cases, most commonly due to desaturation. The remainder of cases were other indications, like altered mental status and sepsis (Table 1). Cross-check of the blood gas analyzer’s internal memory revealed 22 additional ABG samples that were not considered for enrollment during the study periods.

**Table 1:** Characterization of the study population

	*N* (%)
*N*	243
Age (SD)	72.8 (± 17.8)
Female sex	113 (47)
Clinical indication for blood gas assessment	
Respiratory failure	204 (84)
- Desaturation - Suspected/manifest pneumonia - Unknown - Cardiac failure, ROSC - Suspected/manifest PE - Exacerbation of known COPD - Active COVID-19 infection	68 44 40 19 12 11 10
Other	39 (16)
- Altered mental status - Sepsis - Metabolic disorder - Smoke inhalation, airway trauma - Anaphylaxis - Intoxication - Anemia, major bleeding - Hypothermia	13 6 5 4 4 4 2 1
Patient characteristics at the time of sampling	
Heart rate > 90/min	114 (47)
Mean arterial pressure < 65 mmHg	14 (6)
Saturation < 90%	39 (16)
Ongoing supplemental oxygen	71 (29)
Respiratory rate > 20/min	134 (55)
Temperature > 38 or < 36 °C	78 (32)

Median time between venous and arterial sampling was 5 min [IQR 3–9]. For arterial and venous samples, the median time from sampling to analyze was 3 min [IQR 2–5] and 4 min [IQR 2–6], respectively. Mean number of attempts for arterial puncture were 1.2 (SD 0.4) and 1 (SD 0.1) for peripheral venous puncture.

 29% of all patients were on supplemental oxygen by the time of sampling. The mean oxygen flow rate was 5.04 L/min (SD 4.25, min 0.5, max 15) administered through a nasal cannula or oxygen mask. Hypercapnia (paCO_2_ > 45 mmHg) was present in 40 (16%) patients while hypocapnia (paCO_2_ < 35 mmHg) was present in 91 (38%) patients. Signs of hypoxia (paO_2_ < 10 mmHg) were present in 155 (64%) of patients.

### Baseline data

In our dataset, the mean arterial pCO_2_ was 38.3 mmHg (SD 8.96) and venous 45.64 mmHg (SD 9.7). The mean for estimated peCO_2_ was 38.19 mmHg (SD 8.66) for Farkas, 40.87 mmHg (SD 8.88) for Lemoël and 40.84 mmHg (SD 9.7) for Zeserson. Mean differences between venous and arterial pCO_2_ was 7.35 mmHg (SD 5.08, min 0.15, max 29.18). 54% of the collected arterial samples were outside the normal range for pCO_2_ of 35–45 mmHg.

### Conversion models

**Fig. 1:**
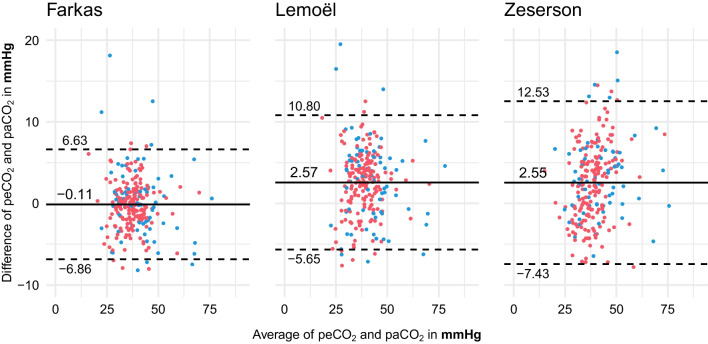
Bland–Altman plots for conversion models of Farkas, Lemoël and Zeserson including all 243 blood gas pairs. *Y*-axis showing the difference between estimated and arterial pCO_2_ in mmHg, mean (black line) and 95% limits of agreement (dashed line). *X*-axis showing average of estimated and arterial pCO_2_ in mmHg. Patients without supplemental oxygen in red, with ongoing supplemental oxygen in blue

For Farkas’ model the mean difference between estimated peCO_2_ and the actual paCO_2_ was − 0.11 mmHg (95% LoA − 6.86, + 6.63; PE 7.2%). For Lemoël’s model the difference was 2.57 mmHg (95% LoA − 5.65, + 10.8; PE 11.9%). For Zeserson’s model the difference between estimated peCO_2_ and the actual paCO_2_ was 2.55 mmHg (95% LoA − 7.43, + 12.53; PE 12.3%) (Fig. 1).

In all three models there was a decrease in precision for patients with ongoing supplemental oxygen therapy (Fig. 1). For Farkas, mean difference was 0.23 mmHg (95% LoA − 8.74, + 9.2; PE 9.6%) for the group with oxygen treatment compared to − 0.26 mmHg (95% LoA − 5.83, + 5.32; PE 6.3%) for the group without supplemental oxygen. The corresponding results for Lemoël were 2.86 mmHg (95% LoA − 7.24, + 12.96; PE 14.3%) and 2.46 mmHg (95% LoA − 4.88, + 9.79; PE 10.9%), respectively, for Zeserson 3.05 mmHg (95% LoA − 8.56, + 14.67; PE 13.4%) and 2.34 mmHg (95% LoA − 6.89, + 11.56; PE 11.8%) (Additional file 1: Fig. S1).

### Subgroup analysis

For the subgroup of patients with a hypercapnic arterial pCO_2_ (> 45 mmHg), the mean difference for Farkas changed to − 2.1 mmHg (95% LoA − 8.56, + 4.36, PE 6.1%), for Lemoël to 0.21 mmHg (95% LoA − 6.75, + 7.18; PE 5.7%) and for Zeserson to 1.59 mmHg (95% LoA − 8.97, + 12.15; PE 8.9%) (Additional file 1: Fig. S2).

For hypocapnic patients (arterial pCO_2_ < 35 mmHg) the mean difference for Farkas changed to − 0.6 mmHg (95% LoA − 6.78, + 7.99; PE 9.9%), for Lemoël 3.44 mmHg (95% LoA − 5.93, + 12.8; PE 17.4%) and for Zeserson 2.87 mmHg (95% LoA − 6.82, + 12.55; PE 15%) (Additional file 1: Fig. S3).

In the logistic regression analysis there was a significant association between the difference of converted pCO_2_ to arterial pCO_2_ and age when adjusting for age, temperature, supplied oxygen, respiratory rate and systolic blood pressure for the Lemoël (OR 0.96, 95%CI 0.93–0.99, *p* = 0.004) and Farkas (OR 0.97, 95%CI 0.95–0.99, *p* = 0.019) models (Additional file 1: Tables S6, S7). There were no significant associations with the Zeserson model (Additional file 1: Table S8). Mean difference and upper limits of agreement were adjusted down for both Farkas and Lemoel but not for Zeserson when looking at patients with an initial desaturation (Additional file 1: Fig. S4) or patients with body temperature above 38 °C (Additional file 1: Fig. S5).

### Confounding factors

**Fig. 2:**
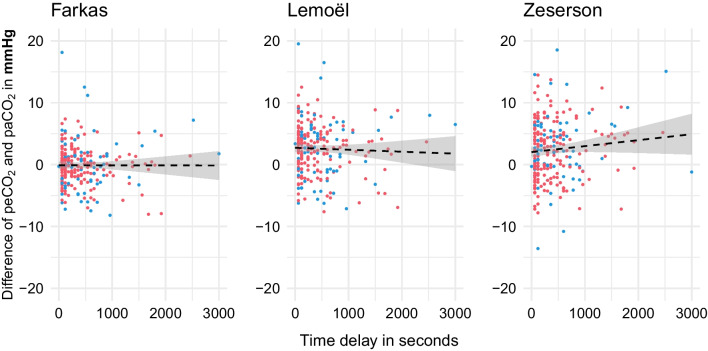
Scatter plots on all three models. *Y*-axis showing the difference between estimated and the arterial pCO_2_ in mmHg with correlation *r* (black dashed line). *X*-axis showing the time delay between venous and arterial sample analysis in seconds. Patients without supplemental oxygen in red, with ongoing supplemental oxygen in blue

There was no significant correlation between the difference for estimated and arterial pCO_2_ and the time delay between venous and arterial sample analysis for any of the models with *r* = − 0.0021 for Farkas (*p* = 0.97), *r* = − 0.037 for Lemoël (*p* = 0.57) and *r* = 0.097 for Zeserson (*p* = 0.13) (Fig. 2).

## Discussion

This is the first external validation of three pCO_2_ conversion models in ED patients with respiratory distress or other indications for blood gas analysis.

Our results show comparable performance for all three models in terms of agreement and precision in estimating true arterial pCO_2_. The adaptive model derived by Farkas showed the overall best metrics with a mean difference of − 0.11 mmHg and 95% limits of agreement of − 6.86 ± 6.63 mmHg for estimated pCO_2_ versus true arterial pCO_2_. Zeserson's model, a commonly used linear model, performed worst with a mean difference between estimated and true arterial pCO_2_ of 2.55 mmHg with wide 95% limits of agreement between 7.43 and + 12.53 mmHg.

In comparison, the unadjusted mean difference between venous and arterial sample was 7.61 mmHg with a standard deviation of 4.7 mmHg. This is comparable to other datasets, the spread in mean difference between different studies is however big and depends on population, setting and studied disease [12–15]. The investigated conversion models performed differently in subgroup analysis, e.g., Lemoëls model showed best agreement for hypercapnic patients with a percentage error of 5.7%, compared to 6.1% (Farkas) and 8.9% (Zeserson). The remaining subgroup analyzes favored the model of Farkas. Variation around the mean is expected for subgroup analysis and since the regression analysis showed no significant association for these variables, and there is limited physiologic rationale around the derivation of coefficients for the models of Lemoel and Farkas, we believe that the results of the subgroups analysis should be interpreted with caution.

Diagnostic accuracy is critical for making informed decisions in critically ill patients, but there is no agreement on what is considered an acceptable precision for estimated pCO_2_ in blood gas analysis.

A prior study of 30 ICU patients showed an inter-sample variation with a 95% confidence interval of 2.4 mmHg for arterial pCO_2_ when blood gasses from both radial arteries were taken simultaneously in the same patient [24]. In another study from Thorson et al. a mean within-patient difference of 3 mmHg (SD 1.9) for pCO_2_ was found in 29 clinically stable ICU patients who underwent six arterial blood gas samplings over 50 min [25]. The mean difference of all models within our study is within this range, although the precision was lower with 95% LoA of − 6.86 to 6.63, − 5.65 to 10.8 and − 7.43 to 12.53 mmHg for Farkas, Lemoël and Zeserson, respectively. Specific clinical situations may still require arterial blood gas sampling but given the known variability in stable patients, a converted venous pCO_2_ is likely sufficient for a considerable share of ED patients.

Since there is currently no consensus on reasonable margins of error for estimated pCO_2_ in critical ill patients, we see a need for further dialogue among ED practitioners on this topic and its effects on clinical decision-making.

From an ICU perspective, the investigated conversion models open up for retrospective pCO_2_ evaluation with reasonable accuracy in critically ill patients initially investigated with venous blood gas sampling in the emergency department.

The administration of supplemental oxygen in the moment of blood gas sampling seemed to cause more outliers in our dataset compared to patients with no supplemental oxygen (Fig. 1). The addition of supplemental oxygen should, to some extent, be accounted for by the SvO_2_ variable for Farkas and Lemoël. The retained mean difference with larger LoA is more likely the result of the smaller sample of the supplemental oxygen group (*n* = 71 vs *n* = 243). Furthermore, when tested in a logistic regression analysis with other potential confounding variables, only age was significantly associated with the difference between arterial and estimated pCO_2_. This was only seen for the Farkas and Lemoël models and the effect was small (OR 0.97 and 0.96, respectively).

As previously shown by Pretto and O’Connor, time delay between sampling and analysis of more than 10 min can falsify analysis results through ingression of room air [19, 26]. In our dataset, the median time to analysis was 3 and 4 min for arterial and venous samples, respectively, and the time difference between venous and arterial sampling was in median 5 min with an IQR of 3 to 9 min. We strived for similar conditions if the clinical situation permitted.

Consequently, there was no influence of time on estimated pCO_2_ when looking at the time difference for Farkas (*R* = 0.013), Lemoël (*R* = − 0.028) or Zeserson (*R* = 0.054), respectively.

In summary, there was a low mean difference between estimated pCO_2_ from venous samples compared to arterial pCO_2_ with better agreement for the adaptive conversion models which is likely sufficient to guide management in most clinical situations in the ED.

### Limitations

Despite the goal of consecutive inclusion there were potentially eligible patients who were not enrolled. Some patients arriving during night hours were missed, primarily due to absence of the enrollment coordinator. However, we did include patients during most night shifts of the study period so it is unlikely that this affected the results in a systematic way.

Arterial blood gas sampling was only indicated in critically ill patients dictated by SOP or as assessed by the treating physician. In spite of that, many patients' vital signs were within normal ranges and the arterial pCO_2_ exceeded normal ranges in only 54% of cases. It is known that low blood pressure, blood loss and other factors compromising circulation in acutely ill patients make the evaluation of pCO_2_ more difficult [27]. A number of patients in a critical state who were directly admitted to intensive care could not be included within 12 h and neither later on. Thus, there may be limited generalizability for patients with more pronounced failure of vital functions. However, this group of patients may require extensive monitoring like an arterial line, removing most of the barriers for arterial blood gas sampling.

This was a single center study using only one type of blood gas analyzer from a single company. Despite the fact that this machine is commonly used in European EDs, we cannot exclude different results with other machines. Neither Lemoël nor Farkas report the blood gas analyzer machines used for analysis of their dataset.

## Conclusion

There was good agreement between estimated venous and arterial pCO_2_ in this study of 243 ED patients using conversion formulas by either Farkas, Lemoël or Zeserson. Thus, conversion of venous pCO_2_ may be considered as an alternative to arterial blood gas sampling with the model of Farkas showing slightly better overall agreement. Additional consideration is required in patients on supplemental oxygen and in patients with hypo- or hypercapnia, both common in critical care and ICU settings.

### Supplementary Information

**Additional file 1**: **Figure S1: **Violin plots on all three models showing the subgroup of supplemental oxygen vs no supplemental oxygen. Y-axis showing subgroups by proportion of total population. X-axis showing the difference between estimated and arterial pCO_2_ in mmHg, mean (black dot) and 95% limits of agreement (black line). The shape of the distribution (skinny on each end and wide in the middle) indicates that data points are highly concentrated around the mean. **Figure S2:** Bland–Altman plots for conversion models of Farkas, Lemoël and Zeserson for the subgroup of patients with a hypercapnic arterial pCO_2_ (> 45 mmHg). Y-axis showing the difference between estimated and arterial paCO_2_ in mmHg, mean (black line) and 95% limits of agreement (dashed line). X-axis showing average of estimated and arterial pCO_2_ in mmHg. Patients without supplemental oxygen in red, with ongoing supplemental oxygen in blue. **Figure S3:** Bland–Altman plots for conversion models of Farkas, Lemoël and Zeserson for the subgroup of patients with a hypocapnic arterial pCO_2_ (< 35 mmHg). Y-axis showing the difference between estimated and arterial paCO_2_ in mmHg, mean (black line) and 95% limits of agreement (dashed line). X-axis showing average of estimated and arterial pCO_2_ in mmHg. Patients without supplemental oxygen in red, with ongoing supplemental oxygen in blue. **Figure S4:** Bland–Altman plots for conversion models of Farkas, Lemoël and Zeserson for the subgroup of patients with initial desaturation (SpO_2_ < 90%). Y-axis showing the difference between estimated and arterial paCO_2_ in mmHg, mean (black line) and 95% limits of agreement (dashed line). X-axis showing average of estimated and arterial pCO_2_ in mmHg. Patients without supplemental oxygen in red, with ongoing supplemental oxygen in blue. **Figure S5:** Bland–Altman plots for conversion models of Farkas, Lemoël and Zeserson for the subgroup of patients with body temperature ≥ 38 °C. Y-axis showing the difference between estimated and arterial paCO_2_ in mmHg, mean (black line) and 95% limits of agreement (dashed line). X-axis showing average of estimated and arterial pCO_2_ in mmHg. Patients without supplemental oxygen in red, with ongoing supplemental oxygen in blue. For comparison: Patients with body temperature lower than 38 °C had a mean difference between estimated peCO_2_ and the actual paCO_2_ for Farkas at -0.07 mmHg (95% LoA -7.15, +7.02), for Lemoël at 2.52 mmHg (95% LoA -6.04, +11.07) and for Zeserson 2.87 mmHg (95% LoA -7.09, +12.83). **Figure S6;** Farkas. **Figure S7:** Lemoël et al. **Figure S8:** Zeserson et al.

## Data Availability

The datasets used and/or analyzed during the current study are available from the corresponding author on reasonable request.
