# Supplementary material for: Agreement of pCO2 in venous to arterial blood gas conversion models in undifferentiated emergency patients
Source: Intensive Care Med Exp. 2023 Nov 21;11:80. doi: 10.1186/s40635-023-00564-w (PMC10663424; doi:10.1186/s40635-023-00564-w)
Supplement: Supplementary file 1 — Additional file 1: Figure S1: Violin plots on all three models showing the subgroup of supplemental oxygen vs no supplemental oxygen. Y-axis showing subgroups by proportion of total population. X-axis showing the difference between estimated and arterial pCO2 in mmHg, mean (black dot) and 95% limits of agreement (black line). The shape of the distribution (skinny on each end and wide in the middle) indicates that data points are highly concentrated around the mean. Figure S2: Bland–Altman plots for conversion models of Farkas, Lemoël and Zeserson for the subgroup of patients with a hypercapnic arterial pCO2 (> 45 mmHg). Y-axis showing the difference between estimated and arterial paCO2 in mmHg, mean (black line) and 95% limits of agreement (dashed line). X-axis showing average of estimated and arterial pCO2 in mmHg. Patients without supplemental oxygen in red, with ongoing supplemental oxygen in blue. Figure S3: Bland–Altman plots for conversion models of Farkas, Lemoël and Zeserson for the subgroup of patients with a hypocapnic arterial pCO2 (< 35 mmHg). Y-axis showing the difference between estimated and arterial paCO2 in mmHg, mean (black line) and 95% limits of agreement (dashed line). X-axis showing average of estimated and arterial pCO2 in mmHg. Patients without supplemental oxygen in red, with ongoing supplemental oxygen in blue. Figure S4: Bland–Altman plots for conversion models of Farkas, Lemoël and Zeserson for the subgroup of patients with initial desaturation (SpO2 < 90%). Y-axis showing the difference between estimated and arterial paCO2 in mmHg, mean (black line) and 95% limits of agreement (dashed line). X-axis showing average of estimated and arterial pCO2 in mmHg. Patients without supplemental oxygen in red, with ongoing supplemental oxygen in blue. Figure S5: Bland–Altman plots for conversion models of Farkas, Lemoël and Zeserson for the subgroup of patients with body temperature ≥ 38 °C. Y-axis showing the difference between estimated and arteria [file 40635_2023_564_MOESM1_ESM.pdf]

Supplementary material:

Agreement of pCO_2 in venous to arterial blood gas conversion models in undifferentiated emergency patients

Matthias Jörg, Malin Öster, Jens Wretborn, Daniel B Wilhelms

Subgroup analysis

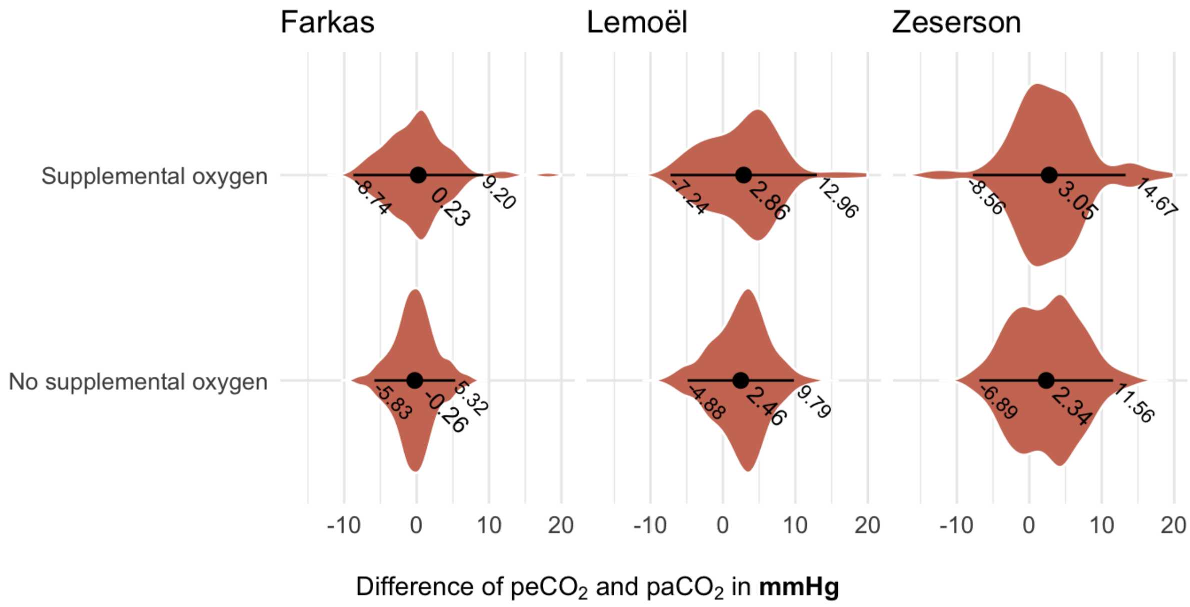

Figure S1: Violin plots on all three models showing the subgroup of supplemental oxygen vs no supplemental oxygen. Y-axis showing subgroups by proportion of total population. X-axis showing the difference between estimated and arterial pCO_2 in mmHg, mean (black dot) and 95% limits of agreement (black line). The shape of the distribution (skinny on each end and wide in the middle) indicates that data points are highly concentrated around the mean.

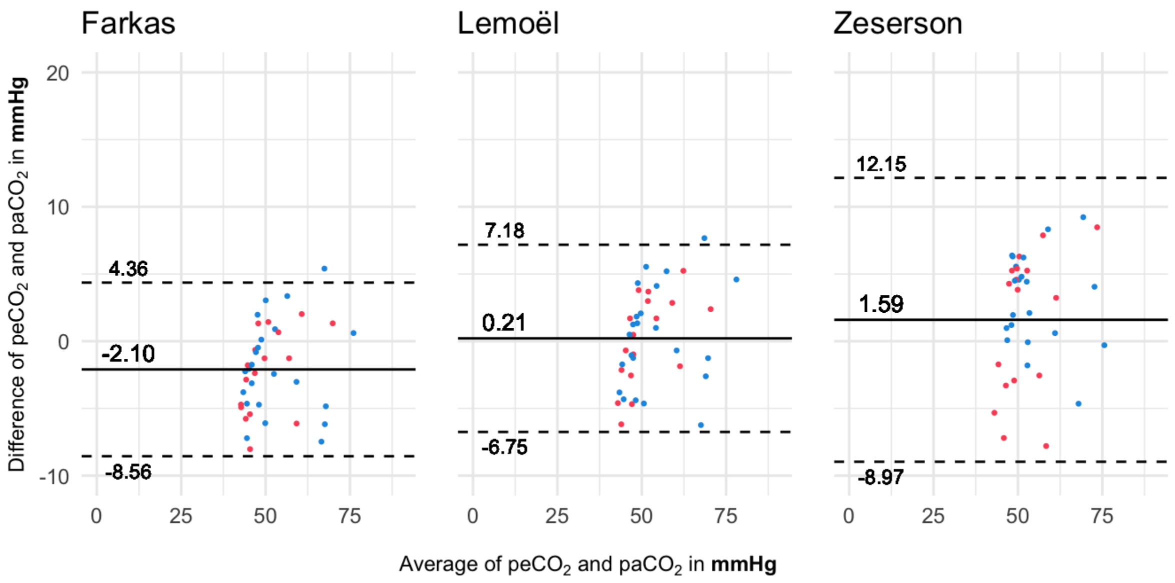

Figure S2: Bland-Altman plots for conversion models of Farkas, Lemoël and Zeserson for the subgroup of patients with a hypercapnic arterial pCO_2 (> 45 mmHg). Y-axis showing the difference between estimated and arterial $paCO_2$ in mmHg, mean (black line) and 95% limits of agreement (dashed line). X-axis showing average of estimated and arterial pCO_2 in mmHg. Patients without supplemental oxygen in red, with ongoing supplemental oxygen in blue.

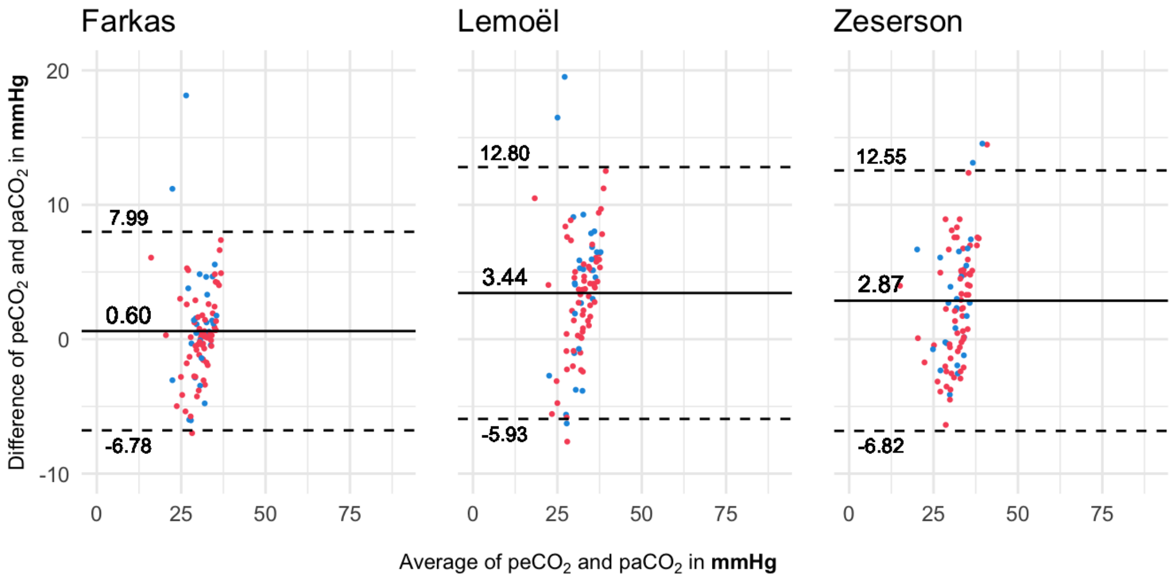

Figure S3: Bland-Altman plots for conversion models of Farkas, Lemoël and Zeserson for the subgroup of patients with a hypocapnic arterial pCO_2 (< 35 mmHg). Y-axis showing the difference between estimated and arterial $paCO_2$ in mmHg, mean (black line) and 95% limits of agreement (dashed line). X-axis showing average of estimated and arterial pCO_2 in mmHg. Patients without supplemental oxygen in red, with ongoing supplemental oxygen in blue.

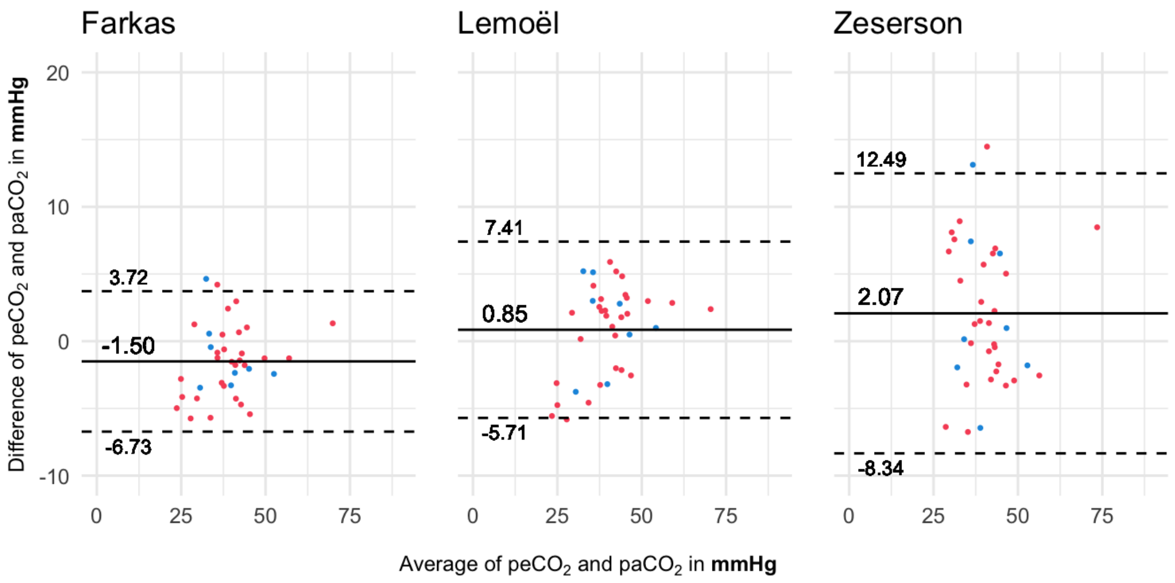

Figure S4: Bland-Altman plots for conversion models of Farkas, Lemoël and Zeserson for the subgroup of patients with initial desaturation (SpO_2 < 90%). Y-axis showing the difference between estimated and arterial $paCO_2$ in mmHg, mean (black line) and 95% limits of agreement (dashed line). X-axis showing average of estimated and arterial pCO_2 in mmHg. Patients without supplemental oxygen in red, with ongoing supplemental oxygen in blue.

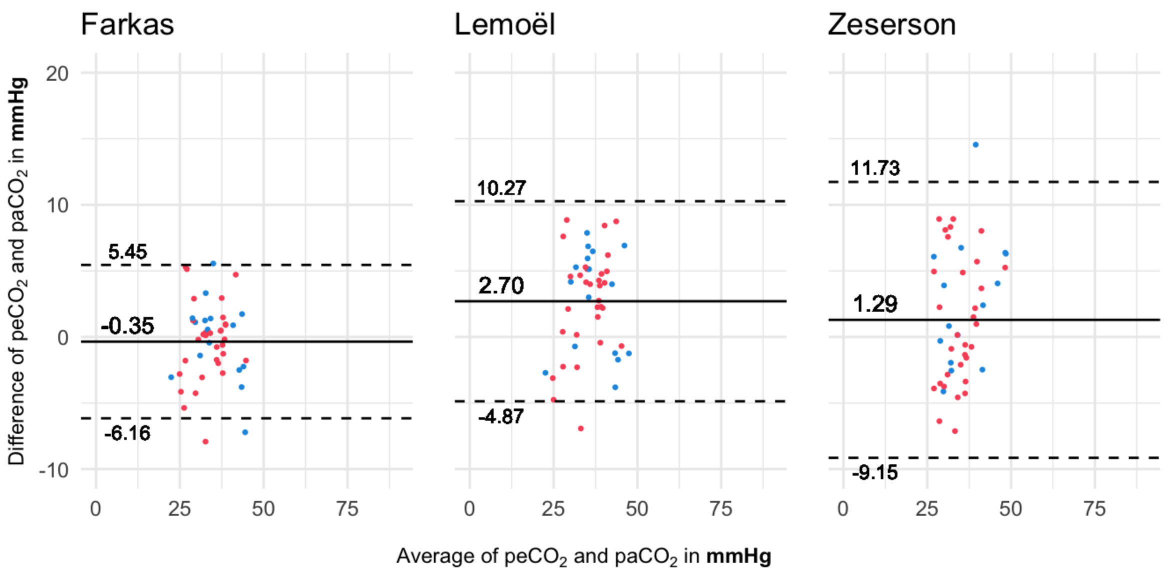

Figure S5: Bland-Altman plots for conversion models of Farkas, Lemoël and Zeserson for the subgroup of patients with body temperature ≥ 38 °C. Y-axis showing the difference between estimated and arterial $paCO_2$ in mmHg, mean (black line) and 95% limits of agreement (dashed line). X-axis showing average of estimated and arterial pCO_2 in mmHg. Patients without supplemental oxygen in red, with ongoing supplemental oxygen in blue. For comparison: Patients with body temperature lower than 38 °C had a mean difference between estimated $peCO_2$ and the actual $paCO_2$ for Farkas at -0.07 mmHg (95% LoA -7.15, +7.02), for Lemoël at 2.52 mmHg (95% LoA -6.04, +11.07) and for Zeserson 2.87 mmHg (95% LoA -7.09, +12.83).

Logistic regression analysis

S6 Farkas

OLS Regression Results						
**Dep. Variable:**	fark_diff	R-squared:	0.032			
**Model:**	OLS	Adj. R-squared:	0.007			
**Method:**	Least Squares	F-statistic:	1.259			
**Date:**	Tue	04 Jul 2023	Prob (F-statistic):	0.277		
**Time:**	17:48:53	Log-Likelihood:	-610.14			
**No. Observations:**	233	AIC:	1234.			
**Df Residuals:**	226	BIC:	1258.			
**Df Model:**	6					
**Covariance Type:**	nonrobust					
	coef	std err	t	P>|t|	[0.025	0.975]
**const**	-2.0004	7.095	-0.282	0.778	-15.980	11.980
**supplied-oxygen**	-0.0222	0.073	-0.305	0.761	-0.166	0.121
**age**	-0.0289	0.012	-2.357	0.019	-0.053	-0.005
**sex**	-0.5347	0.443	-1.206	0.229	-1.409	0.339
**systolic-bp**	-0.0002	0.008	-0.029	0.977	-0.016	0.016
**respiratory-rate**	0.0052	0.041	0.125	0.900	-0.076	0.087
**temperature**	0.1068	0.185	0.578	0.564	-0.257	0.471
**Omnibus:**	43.645	Durbin-Watson:	1.910			
**Prob(Omnibus):**	0.000	Jarque-Bera (JB):	154.495			
**Skew:**	0.715	Prob(JB):	2.83e-34			
**Kurtosis:**	6.724	Cond. No.	5.07e+03			

S7 Lemoël et al.

OLS Regression Results						
**Dep. Variable:**	lemoel_pco2_diff	R-squared:	0.050			
**Model:**	OLS	Adj. R-squared:	0.024			
**Method:**	Least Squares	F-statistic:	1.965			
**Date:**	Tue	04 Jul 2023	Prob (F-statistic):	0.0717		
**Time:**	17:48:53	Log-Likelihood:	-657.48			
**No. Observations:**	233	AIC:	1329.			
**Df Residuals:**	226	BIC:	1353.			
**Df Model:**	6					
**Covariance Type:**	nonrobust					
	coef	std err	t	P>|t|	[0.025	0.975]
**const**	-5.9287	8.693	-0.682	0.496	-23.058	11.201
**supplied-oxygen**	-0.0374	0.089	-0.419	0.675	-0.213	0.138
**age**	-0.0443	0.015	-2.942	0.004	-0.074	-0.015
**sex**	-0.4742	0.543	-0.873	0.384	-1.545	0.597
**systolic-bp**	0.0006	0.010	0.056	0.956	-0.019	0.020
**respiratory-rate**	0.0135	0.051	0.267	0.790	-0.086	0.113
**temperature**	0.3111	0.226	1.375	0.171	-0.135	0.757
**Omnibus:**	8.122	Durbin-Watson:	1.942			
**Prob(Omnibus):**	0.017	Jarque-Bera (JB):	13.187			
**Skew:**	0.147	Prob(JB):	0.00137			
**Kurtosis:**	4.128	Cond. No.	5.07e+03			

OLS Regression Results						
**Dep. Variable:**	zeserson_diff	R-squared:	0.025			
**Model:**	OLS	Adj. R-squared:	-0.001			
**Method:**	Least Squares	F-statistic:	0.9730			
**Date:**	Tue	04 Jul 2023	Prob (F-statistic):	0.444		
**Time:**	17:48:53	Log-Likelihood:	-699.35			
**No. Observations:**	233	AIC:	1413.			
**Df Residuals:**	226	BIC:	1437.			
**Df Model:**	6					
**Covariance Type:**	nonrobust					
	coef	std err	t	P>|t|	[0.025	0.975]
**const**	21.1846	10.404	2.036	0.043	0.683	41.686
**supplied-oxygen**	0.0234	0.107	0.219	0.827	-0.187	0.234
**age**	0.0170	0.018	0.946	0.345	-0.018	0.053
**sex**	-0.7162	0.650	-1.101	0.272	-1.998	0.565
**systolic-bp**	-0.0026	0.012	-0.218	0.827	-0.026	0.021
**respiratory-rate**	-0.0198	0.061	-0.327	0.744	-0.139	0.100
**temperature**	-0.5060	0.271	-1.869	0.063	-1.040	0.028
**Omnibus:**	10.966	Durbin-Watson:	1.864			
**Prob(Omnibus):**	0.004	Jarque-Bera (JB):	13.492			
**Skew:**	0.377	Prob(JB):	0.00118			
**Kurtosis:**	3.906	Cond. No.	5.07e+03			

Values in kPa

Unadjusted baseline data

In our dataset, the mean arterial pCO_2 was 5.1 kPa (SD 1.2) and venous 6.09 kPa (SD 1.3). Mean differences between venous and arterial pCO_2 was 0.98 kPa (SD 0.68, min 0.02, max 3.89). 54% of the collected arterial samples were outside the normal range for pCO_2 of 4.7 – 6 kPa.

Conversion Models

For Farkas' model the mean difference between estimated $peCO_2$ and the actual $paCO_2$ was -0.04 kPa (95% LoA -0.94, +0.87). For Lemoël's model the difference was 0.34 kPa (95% LoA -0.75, +1.44). For Zeserson's model the difference between estimated $peCO_2$ and the actual $paCO_2$ was 0.34 kPa (95% LoA -0.99, +1.67).

In all three models there was a decrease in precision for the subgroup of patients with ongoing supplemental oxygen therapy. For Farkas, mean difference was 0.01 kPa (95% LoA -1.19, +1.21) for the group with oxygen treatment compared to -0.06 kPa (95% LoA -0.81, +0.70) for the group without supplemental oxygen. The corresponding results for Lemoël were 0.38 kPa (95% LoA -0.97, +1.73) and 0.33 kPa (95% LoA -0.65, +1.31), respectively, for Zeserson 0.41 kPa (95% LoA -1.14, +1.96) and 0.31 kPa (95% LoA -0.92, +1.54).
